# Intercropping with potato-onion alters arbuscular mycorrhizal fungi spore-associated bacterial communities of tomato rhizosphere

**DOI:** 10.3389/fmicb.2025.1686962

**Published:** 2025-11-26

**Authors:** Liyan Zhou, Xinjie Pan, Zihan Zhang, Ying Zhang, Fengzhi Wu, Danmei Gao

**Affiliations:** 1 Key Laboratory of Biology and Genetic Improvement of Horticultural Crops (Northeast Region), Ministry of Agriculture and Rural Affairs, Northeast Agricultural University, Harbin, China; 2 Department of Horticulture, Northeast Agricultural University, Harbin, China; 3 Department of Resources and Environment, Northeast Agricultural University, Harbin, China

**Keywords:** intercropping, tomato, potato-onion, AMF spore-associated bacteria, bacterial community

## Abstract

Intercropping systems optimize soil ecological functions, modulate microbial diversity, and enhance crop productivity. Arbuscular mycorrhizal fungi (AMF) are key soil symbionts that facilitate nutrient acquisition and enhance stress resilience in host plants. Notably, the AMF spore-associated bacterial communities that play a key role in maintaining AMF spore viability and supporting AMF function remain understudied in intercropping systems. This knowledge gap limits our ability to optimize intercropping's ecological benefits (e.g., enhanced soil fertility, reduced reliance on chemical fertilizers) by leveraging plant-AMF-bacteria synergies, which are critical for sustainable agriculture. This study compared the effects of tomato (*Solanum lycopersicum* L.) monocropping vs. tomato/potato-onion (*Allium cepa* L. var. *aggregatum* G. Don) intercropping on the composition and diversity of AMF spore-associated bacterial communities in the tomato rhizosphere under controlled greenhouse conditions, using Illumina MiSeq sequencing of the 16S rRNA gene V3–V4 region. The results demonstrated that compared with tomato monocropping, tomato/potato-onion intercropping significantly increased the alpha diversity (Shannon and Chao1 indices) of AMF spore-associated bacterial communities in the tomato rhizosphere (Student's *t*-test, *P* < 0.05) and markedly altered their taxonomic composition. Taxa significantly enriched under the intercropping system included the phyla Actinobacteriota and Cyanobacteria, the classes Alphaproteobacteria and Actinobacteria (a class of phylum Actinobacteriota), and the genera *Janthinobacterium, Rhodococcus, Paenarthrobacter*, and *Streptomyces*. Differential analysis identified 156 significantly shifted OTUs, with 137 enriched (predominantly *Proteobacteria*/*Actinobacteria*) and 19 depleted (mostly *Bacteroidetes*/*Proteobacteria*) in intercropping. These findings demonstrate that tomato/potato-onion intercropping reshapes AMF spore-associated microbiomes, selectively enriching microbial taxa with putative functions in nutrient cycling and plant growth promotion.

## 1 Introduction

Intercropping, a traditional agricultural practice globally, optimizes crop canopy structure, enhances the efficient utilization of natural resources, and exerts positive impacts on crop productivity and the soil microbial environment (Nyawade et al., 2019). Compared with monoculture, intercropping confers distinct ecosystem benefits by modulating soil microbial communities (Wang et al., 2018), particularly those closely associated with key symbiotic fungi. Specifically, Gao D. M. et al. (2021) demonstrated that tomato intercropped with potato-onion significantly promotes tomato growth, improves nutrient uptake, and increases the abundance of rhizosphere arbuscular mycorrhizal fungal (AMF) communities. This tomato/potato-onion intercropping system functions as a beneficial companion cropping strategy, but the bacteria associated with AMF spores—a group directly regulating AMF spore germination and early symbiosis establishment (Chu et al., 2022), which are critical for sustaining AMF's nutrient cycling function—remain uncharacterized in this system.

Arbuscular mycorrhizal fungi (AMF) are core components of the “rhizospheric symbiotic community” (Rhizobiont), serving as key symbionts that mobilize insoluble nutrients, enhance mineral uptake, and promote plant growth (Brundrett and Tedersoo, 2018; Wang and Qiu, 2006; Wang et al., 2024). The ecological functions of AMF are highly dependent on their associated microorganisms: AMF mycelial exudates recruit specialized bacterial communities in the hyphosphere (Zhang et al., 2022), while bacteria colonizing the surfaces or interiors of AMF spores form a unique symbiotic microenvironment (Artursson and Jansson, 2003; Bianciotto et al., 2001). These AMF spore-associated bacteria differ functionally from rhizospheric or hyphospheric bacteria—unlike rhizospheric bacteria, which primarily interact with plant roots, or hyphospheric bacteria, which associate with extended mycelia, spore-associated bacteria directly regulate AMF spore germination, viability, and the establishment of initial symbiosis (Chu et al., 2022; Toljander et al., 2006). For example, specific bacterial taxa can increase AMF spore germination rates and support early mycelial growth, a process that is foundational for the subsequent formation of plant-AMF symbiosis and the execution of nutrient cycling functions (Bidondo et al., 2016; Duan et al., 2024).

Despite the importance of AMF spore-associated bacteria, current research on intercropping and soil microorganisms has primarily focused on rhizosphere microbial communities or AMF themselves (Van der Heijden and Wagg, 2013; Jain et al., 2020). Studies have shown that, compared with conventional tillage, intercropping increases the abundance and diversity of soil microbial communities in orchard systems (Lacombe et al., 2009); however, how intercropping regulates AMF spore-associated bacterial communities—particularly in the tomato/potato-onion system—remains largely unknown. This knowledge gap limits our understanding of the “plant-AMF-bacteria” synergy mechanisms that underpin the benefits of intercropping, thereby hindering efforts to optimize intercropping practices for enhanced soil fertility and sustainable agricultural production.

In the present study, we compared the effects of tomato monoculture and tomato/potato-onion intercropping on AMF spore-associated bacterial communities in the tomato rhizosphere. Using Illumina MiSeq sequencing, we analyzed the abundance, diversity, and taxonomic composition of these bacterial communities. Our primary objective was to investigate how interspecific plant interactions impact the diversity and structural composition of AMF spore-associated microbiomes. To address these knowledge gaps, we posed the following research questions: (1) Does tomato/potato-onion intercropping alter the alpha diversity of AMF spore-associated bacterial communities relative to monoculture? (2) Does intercropping enrich specific bacterial taxa linked to plant-beneficial potential (e.g., nutrient cycling, growth promotion)?

## 2 Materials and methods

### 2.1 Experimental design

This study was conducted in a greenhouse at the Horticultural Research Station of Northeast Agricultural University, Harbin, China (45°41′N, 126°37′E). Test soil was collected from the top 0-15 cm layer of a low-phosphorus open field at the Xiangyang Research Base in Harbin, where native vegetation has been growing for over 20 years. The soil is classified as black soil with a sandy loam texture, and its basic chemical properties are as follows: organic matter, 2.92%; inorganic nitrogen (${\text{NH}}_{4}^{-}$-N and ${\text{NO}}_{3}^{+}$-N), 134.19 mg kg^−1^; available potassium (AK), 70.19 mg kg^−1^; available phosphorus (AP), 14.53 mg kg^−1^; electrical conductivity (EC, 1:5, w/v), 0.25 mS cm^−1^; pH (1:5, w/v), 6.61; and AMF spore numbers were ca. 500 per 100 g of the soil (Gerdemann and Nicolson, 1963).

The plant species selected for this study included tomato and potato-onion, with the tomato cultivar specified as “Dongnong 708” (*Solanum lycopersicum* L.) and the potato-onion cultivar identified as “Suihua” (*Allium cepa* L. var. *aggregatum* G. Don). Both are locally adapted varieties widely used in agricultural research and production within the study region, and all plant materials were provided by the Key Laboratory of Horticultural Crop Biology and Genetic Improvement, Ministry of Agriculture and Rural Affairs, Northeast Agricultural University.

Two experimental treatments were established: a tomato monoculture system (T) and a tomato/potato-onion intercropping system (TO). The experiment was conducted in plastic pots (22.5 cm in diameter × 14.0 cm in height) (Wu et al., 2019), which were thoroughly cleaned and sterilized with 75% ethanol prior to use to eliminate potential microbial contaminants; each pot contained 3 kg of soil. A randomized complete block design was adopted, with three replicates per treatment and 15 pots per replicate. Tomato seeds were surface-sterilized with 3.8% sodium hypochlorite and germinated in a sterilized mixture of sand and low-P soil (1:1, v/v). After 15 days, tomato seedlings with two true leaves were transplanted. Potato-onion bulbs were stored at 4 °C before planting. For the tomato monoculture system (T), two uniformly sized tomato seedlings at the two-true-leaf stage were transplanted into each pre-prepared pot. The two seedlings were planted approximately 5 cm apart in a parallel arrangement to ensure balanced access to resources. In the tomato/potato-onion intercropping system (TO), one tomato seedling and three potato-onion bulbs were planted per pot. The potato-onion bulbs were placed 5–10 cm away from the tomato seedling in the horizontal direction, corresponding to a planting ratio of 3:1 (potato-onion:tomato). This arrangement ensured sufficient space for root development while facilitating potential interspecific interactions between the two species.

Therefore, the experiment comprised 2 cropping systems (monoculture and intercropping) × 3 replicates × 15 pots, totaling 90 pots. During sample analysis, a subsampling strategy was implemented to ensure representativeness: 5 pots were randomly selected from the 15 pots of each biological replicate, and their rhizosphere soils were pooled to form one composite sample per replicate, resulting in 3 composite samples per treatment for subsequent analyses. Soil moisture in all pots was maintained at 70% of the soil's water-holding capacity by adding groundwater every 1–3 days, ensuring optimal plant growth conditions. Weeds were manually removed throughout the experiment. To prevent the leaching of soil microorganisms with irrigation water, the inner walls of the plastic pots were lined with plastic film.

### 2.2 Soil sampling

Rhizosphere soil sampling was conducted 90 days after transplantation following this specific protocol: Five tomato plants were randomly selected from each replicate of each treatment. The roots were gently shaken, and rhizosphere soil was collected using a sterile bristle brush. Soil samples from each replicate were then thoroughly homogenized and sieved through a 2 mm mesh to remove impurities. This yielded three composite soil samples per treatment, which were stored at 4 °C for subsequent extraction of AMF spore-associated bacteria.

### 2.3 Preparation of AMF spore-associated bacterial suspension

AMF spores in the rhizosphere soil samples were extracted using the wet sieving and sucrose density gradient centrifugation method (Daniels and Skipper, 1982). The soil samples were first mixed with sterile water at a ratio of 1:5 (w/v) and shaken for 10 min to fully disperse the spores. The sucrose density gradient used was 10%−50% (w/v), with centrifugation performed at 3000 × g for 20 min at 4 °C, and AMF spores were collected from the interface layer (Rillig, 2004). The extracted spores were further identified as AMF spores via microscopic examination, based on typical morphological characteristics such as spherical or elliptical shape, thick walls, possible surface ornamentation, and non-septate sporangiophores, to exclude spores of other fungi (Johnson et al., 2003).

The AMF spore suspension obtained after extraction was subjected to AMF spore retention using a 0.45 μm aqueous filter membrane. Subsequently, the filter membrane is placed in a centrifuge tube and washed vigorously with a vortex mixer at 1,500 rpm for 1 min to obtain a suspension of AMF spore symbiotic bacteria (Agnolucci et al., 2015). This bacterial suspension was stored at 4 °C for no more than 48 h for subsequent isolation and purification (Brundrett and Tedersoo, 2018). For long-term preservation, 20% glycerol (final concentration) was added, and the suspension was stored at−80°C (Stürmer et al., 2021).

### 2.4 Illumina MiSeq sequencing

DNA extraction and Illumina MiSeq sequencing of AMF spore-associated bacteria were performed by Shenggong Bioengineering (Shanghai) Co., Ltd. Total genomic DNA was extracted from the bacterial suspension using a TIANamp Bacteria DNA Kit (TIANGEN, China) following the manufacturer's protocol; DNA quality was verified via 1% agarose gel electrophoresis, and concentration was determined using a NanoDrop spectrophotometer (Thermo Fisher Scientific, USA) with ${\text{OD}}_{2}^{60}$/${\text{OD}}_{2}^{80}$ ratios maintained between 1.8–2.0.

The V3–V4 hypervariable regions of the bacterial 16S rRNA gene were amplified using the primer pair F338/R806, which is widely used for rhizosphere microbial community analysis due to its high specificity (Zhou et al., 2018). Each forward and reverse primer was tagged with a unique 6-bp barcode for sample-specific discrimination. PCR reactions were conducted in 25 μL volumes containing 12.5 μL 2 × Taq PCR MasterMix (CWBIO, China), 1 μL of each primer (10 μM), 2 μL template DNA, and 8.5 μL sterile ddH_2_O. The thermal profile was: 95 °C for 3 min (initial denaturation); 35 cycles of 95 °C for 30 s, 55 °C for 30 s, and 72 °C for 45 s; and a final extension at 72 °C for 10 min.

For each composite DNA sample, three technical replicates of PCR amplification were performed. Amplicons from the replicates were pooled, purified using an Agarose Gel DNA Purification Kit (TaKaRa, Dalian, China), and quantified with a Qubit 4 Fluorometer (Thermo Fisher Scientific, USA). Purified products were pooled in equimolar concentrations to construct the sequencing library, which was quality-checked using an Agilent 2100 Bioanalyzer (Agilent Technologies, USA) to confirm target fragment sizes (~460 bp) before Illumina MiSeq PE300 sequencing.

### 2.5 Raw sequence data processing

The raw sequence files were quality-filtered and processed using FLASH following the methods described previously (Magoč and Salzberg, 2011; Gao et al., 2017). Chimeric sequences were identified and removed using USEARCH 6.1 implemented in QIIME (Caporaso et al., 2010). Operational taxonomic units (OTUs) were clustered at 97% sequence similarity using UPARSE with an agglomerative clustering algorithm (Edgar, 2013). The representative sequence of each operational taxonomic unit (OTU) was taxonomically annotated against the SILVA database (release 128; Quast et al., 2012; Kõljalg et al., 2013). The raw sequences have been uploaded to the NCBI Sequence Read Archive (accession number PRJNA1291378).

### 2.6 Statistical analysis

Alpha diversity indices (Chao1, Shannon, Simpson) were calculated in QIIME 1 to evaluate community richness and evenness. Beta diversity was analyzed via principal coordinates analysis (PCoA) based on Bray-Curtis dissimilarity to assess overall compositional differences. Heatmap analysis of dominant taxa abundance patterns was performed using the “pheatmap” package in R (Kolde, 2015). Differences in the relative abundance of OTUs between treatments were measured using likelihood ratio tests with the Benjamini-Hochberg *p* value correction in the “EdgeR” package (Robinson et al., 2010). Volcano plots (via “ggpubr” and “ggthemes” in R) visualized differential OTU magnitude and significance, while Manhattan plots (via “ggplot2” in R) displayed taxonomic distribution of significant differences. Alpha diversity indices and taxonomic compositions (phylum, class, OTU levels) of AMF spore-associated bacteria were compared between monoculture and intercropping systems using Student's *t*-test (*P* < 0.05). Statistical analyses including Student's *t*-test were conducted in IBM SPSS Statistics v22.0 (IBM Corp., Armonk, NY, USA); analyses involving EdgeR, PCoA, and heatmaps were performed in R.

## 3 Results

### 3.1 Illumina Miseq sequencing data

In total, Illumina MiSeq sequencing generated 294,606 high-quality bacterial sequences, with individual samples containing 36,182–63,878 sequences (mean = 49,101). The 16S rRNA gene amplicons average read length was 423 bp. Good's coverage, an indicator of captured community diversity, exceeded 99.88 ± 0.08% across all bacterial communities, demonstrating comprehensive sampling of the dominant taxa. Rarefaction curves for OTUs clustered at 97% sequence similarity approached saturation for all samples (data not shown), confirming that the sequencing depth was sufficient to accurately assess bacterial community diversity.

### 3.2 Diversity of AMF spore-associated bacterial communities

**Figure 1:**
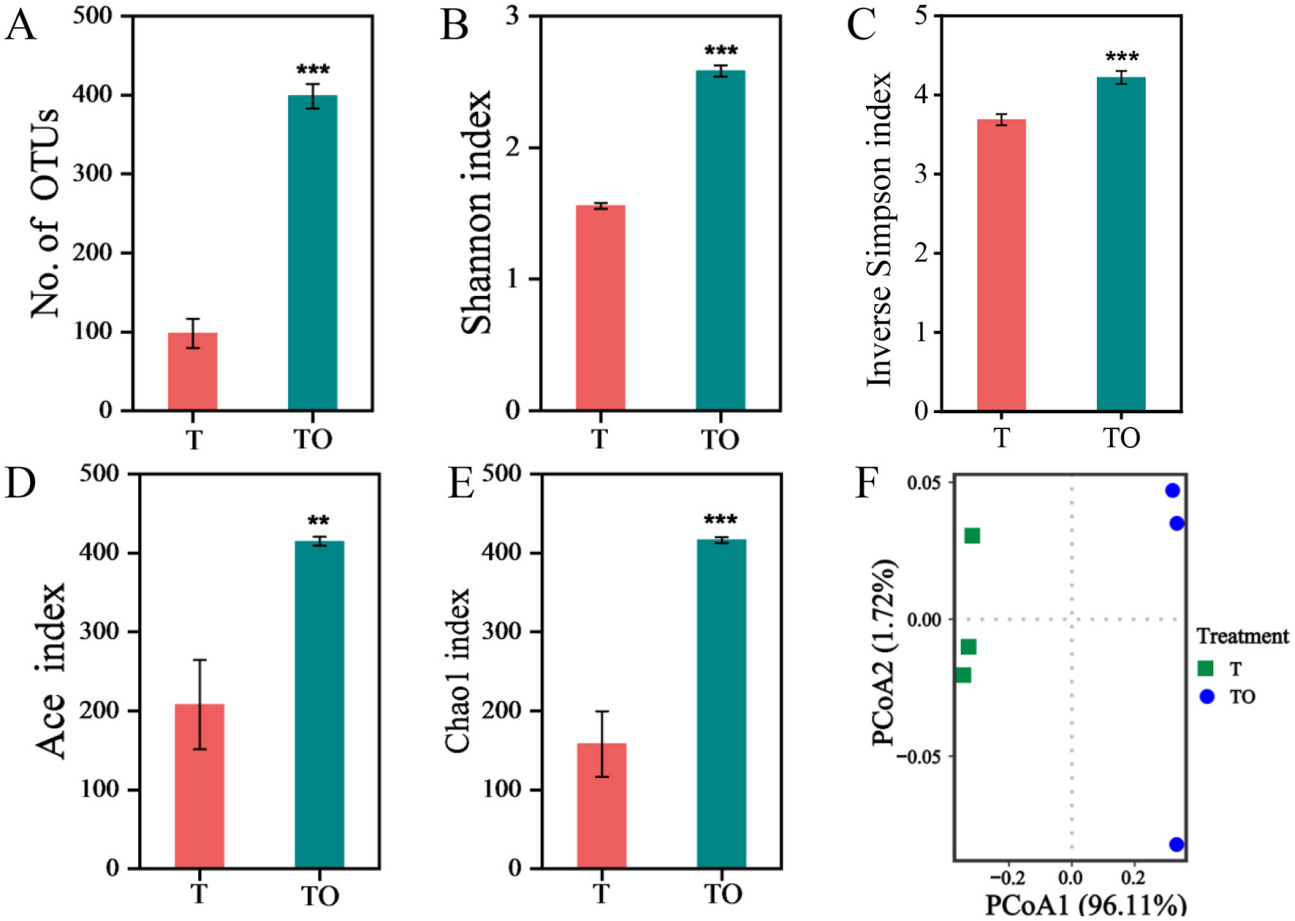
Alpha **(A–E)** and beta **(F)** diversity of AMF spore-associated bacterial communities in tomato monoculture (T) and tomato/potato-onion intercropping (TO) systems. Beta diversity was analyzed based on Bray-Curtis dissimilarity at the OTU level (97% sequence similarity). ** and *** indicate significant difference at *P* < 0.01, and *P* < 0.001, respectively (Student's *t*-test).

Alpha diversity of AMF spore-associated bacterial communities in treatments T and TO was quantified using OTU richness, Shannon Index, inverse Simpson Index, ACE Index, and Chao1 Index. All assessed alpha diversity metrics were significantly elevated in the TO system compared to the T treatment (*P* < 0.05), indicating a higher level of community richness and evenness in the intercropping system (Figures 1A–E). At the OTU level, principal coordinate analysis (PCoA) revealed a distinct clustering pattern, with clear separation in community composition of AMF spore-associated bacteria between monoculture (T) and intercropping (TO) systems (Figure 1F).

### 3.3 Composition of AMF spore-associated bacterial communities

**Figure 2:**
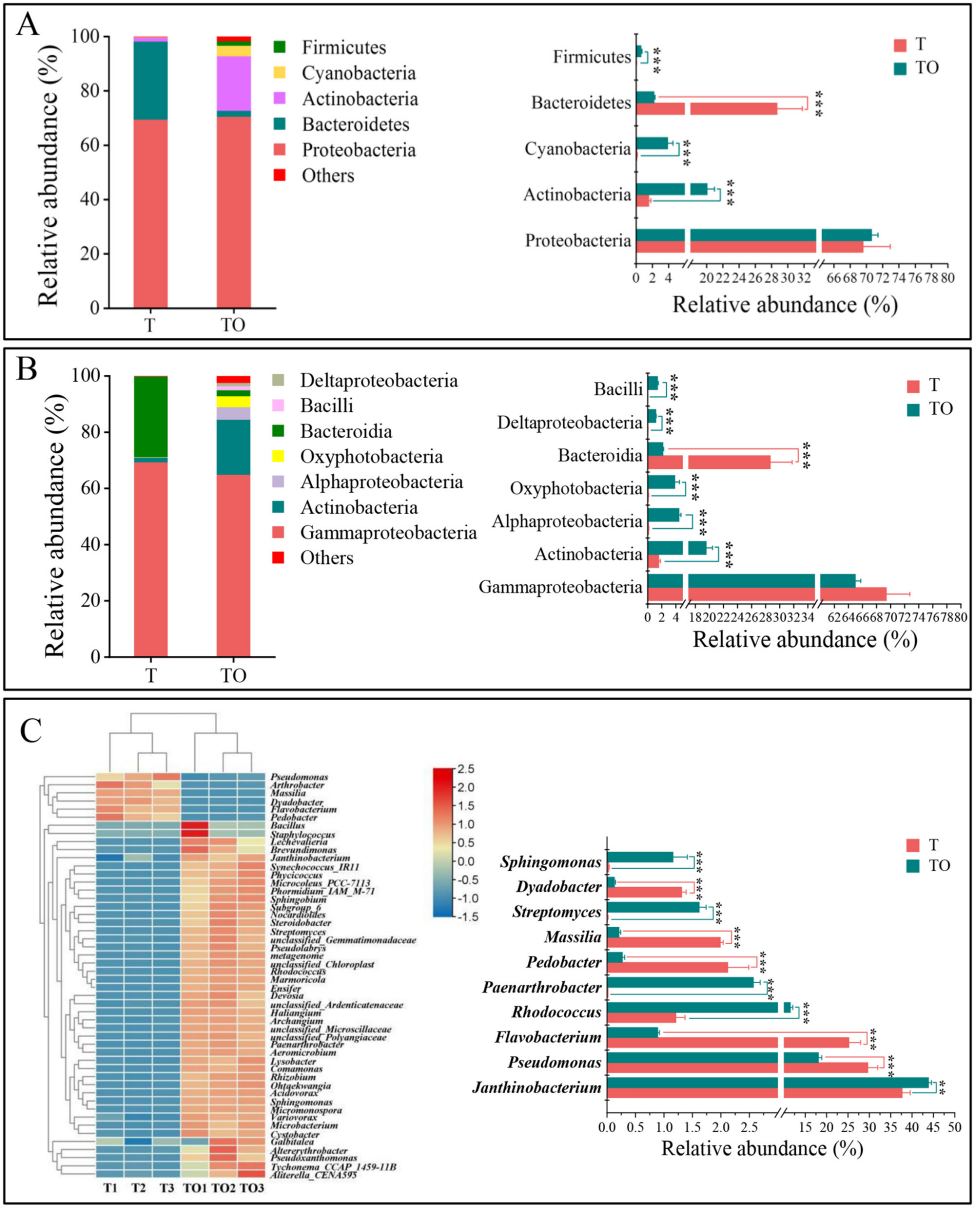
Composition of AMF spore-associated bacterial communities at the phylum **(A)** and class **(B)** levels in tomato monoculture (T) and tomato/potato-onion intercropping (TO) systems. **(A, B)** Relative abundances of dominant bacterial phyla and classes (with relative abundances > 1% in at least one treatment). **(C)** Heatmap of the top 50 genera of AMF spore-associated bacterial communities under different treatments. In the heatmap, relative abundances of bacterial genera in T and TO are represented by a color gradient from red (high abundance) to yellow to blue (low abundance). Hierarchical clustering of treatments was performed using the average linkage method based on Euclidean distances. For differential analysis in the heatmap, only bacterial taxa with relative abundances > 1% in at least one treatment were included for further comparison. Statistical significance is indicated by *, **, and *** for *P* < 0.05, *P* < 0.01, and *P* < 0.001, respectively, based on Student's *t*-test.

At the phylum level, a total of 15 bacterial phyla were identified across all samples (data not shown). Among these, Proteobacteria, Bacteroidetes, and Actinobacteriota emerged as the core dominant phyla, collectively contributing to over 92% of the total bacterial sequence reads (average relative abundance >1%) (Figure 2A). Comparative analysis revealed that the intercropping system (TO) exhibited significantly higher relative abundances of Actinobacteriota, Cyanobacteria, and Firmicutes compared to the monoculture system (T), whereas the relative abundance of Bacteroidetes was significantly decreased in TO (*P* < 0.05) (Figure 2A).

At the class level, 44 distinct bacterial classes were detected across all samples (data not shown). Gammaproteobacteria, Actinobacteria, and Bacteroidia represented the dominant taxonomic classes, accounting for more than 86% of the total sequence reads (average relative abundances >1%) (Figure 2B). Relative to T, the TO system displayed significantly elevated abundances of Actinobacteria, Alphaproteobacteria, Oxyphotobacteria, Bacilli, and Deltaproteobacteria, while the relative abundance of Bacteroidia was significantly reduced in TO (*P* < 0.05) (Figure 2B).

At the genus level, 274 bacterial genera were identified in total (data not shown), with the top 50 genera based on relative abundance visualized in a heatmap (Figure 2C). Specifically, the relative abundances of *Janthinobacterium, Rhodococcus, Paenarthrobacter, Streptomyces, Sphingomonas, Bacillus, Microbacterium, Phormidium* IAM M-71, *Tychonema* CCAP 1459-11B, *Aeromicrobium, Micromonospora, Acidovorax, Allorhizobium, Microcoleus* PCC 7113, *Staphylococcus, Nocardioides, Marmoricola, Haliangium, Devosia*, and *Sphingobium* were significantly higher in TO than in T. Conversely, the relative abundances of *Pseudomonas, Flavobacterium, Pedobacter, Massilia*, and *Dyadobacter* were significantly lower in the TO system (*P* < 0.05) (Figure 2C, Supplementary Table 1). Notably, the tomato/potato-onion intercropping system exerted a specific regulatory effect on the abundances of *Microcoleus* PCC 7113, *Staphylococcus, Comamonas*, and *Ensifer* (Supplementary Table S1). Notably, enriched genera such as *Streptomyces* (known for secondary metabolite production) and *Bacillus* (plant growth promoters) suggest potential functional shifts toward enhanced nutrient mobilization and symbiotic efficiency in intercropping (Figure 2C, Supplementary Table 1).

**Figure 3:**
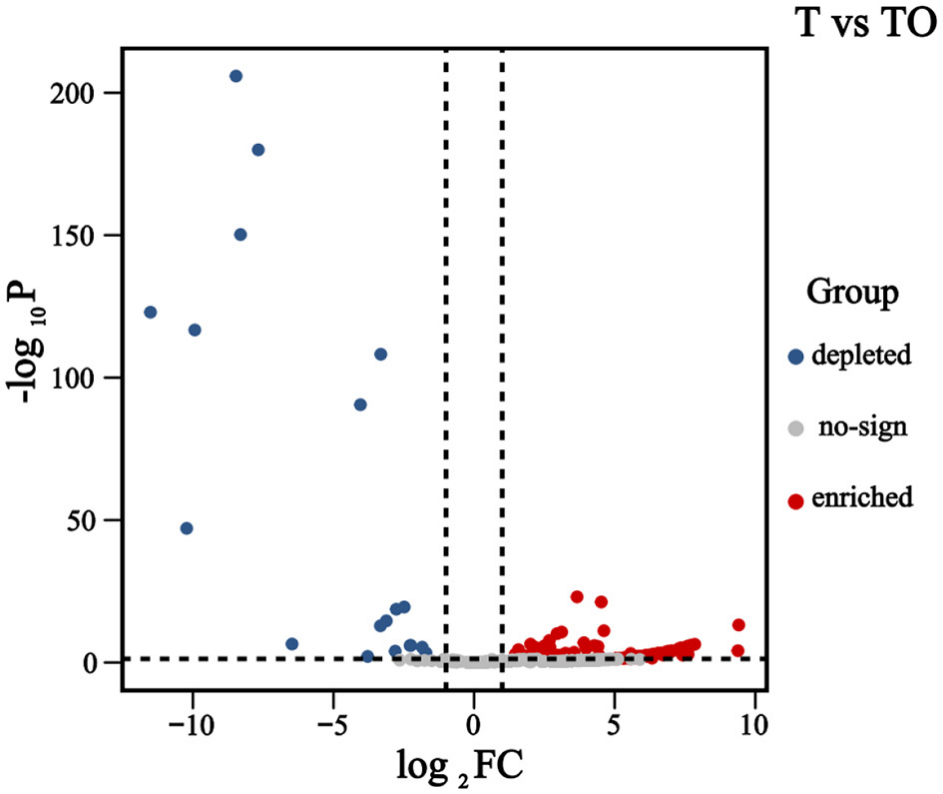
Volcano plot illustrating differentially abundant OTUs between cropping systems. Notes: Each point in the volcano plot represents an individual OTU. The X-axis denotes the log_2_ fold change (log_2_FC) in OTU abundance between treatments, while the Y-axis represents the negative logarithm of the adjusted *P*-value (-log_10_ adjusted *P*-value), indicating the statistical significance of abundance changes. A larger absolute value on the X-axis indicates a greater magnitude of difference in OTU abundance between groups, whereas a higher Y-axis value reflects a more significant difference. Red dots represent significantly enriched OTUs in the intercropping system (TO), blue dots represent significantly depleted OTUs in TO, and gray dots indicate OTUs with no significant difference (*P* ≥ 0.05 or |log _2_ FC| ≤ 1).

A total of 443 bacterial operational taxonomic units (OTUs) were detected within AMF spores (Figure 3). Compared with the monoculture system (T), 137 OTUs were significantly enriched in the intercropping system (TO); these enriched OTUs were predominantly affiliated with the phyla Proteobacteria, Actinobacteriota, and Cyanobacteria. In contrast, 19 OTUs were significantly depleted in TO relative to T, and the majority of these depleted OTUs belonged to the phyla Bacteroidetes and Proteobacteria.

**Figure 4:**
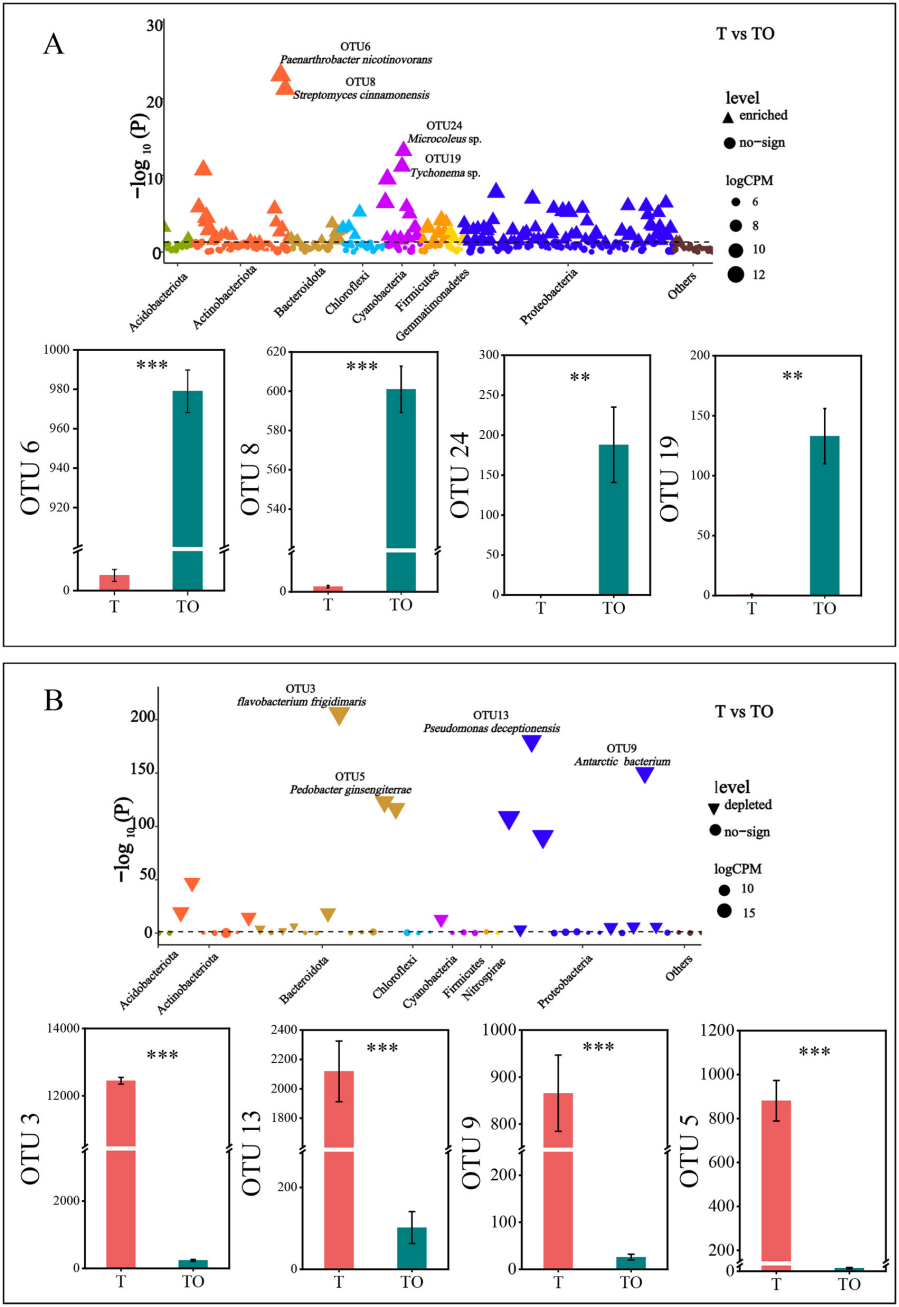
Manhattan plots and associated bar plots showing differentially abundant OTUs between cropping systems. **(A)** Manhattan plot displaying taxonomic information for OTUs enriched in the tomato/potato-onion intercropping system (TO) relative to the tomato monoculture system (T), with a corresponding bar plot showing counts of representative enriched OTUs (OTU6, OTU8, OTU24, OTU19). **(B)** Manhattan plot presenting taxonomic information for OTUs depleted in TO compared to T, accompanied by a bar plot showing counts of representative depleted OTUs (OTU3, OTU13, OTU9, OTU5). The dashed line indicates the false discovery rate (FDR)-corrected significance threshold (*P* = 0.05). ** and *** indicate significant differences at *P* < 0.01 and *P* < 0.001, respectively (Student's *t*-test). CPM = counts per million; OTU = operational taxonomic unit.

The most significantly enriched OTUs included OTU6 (8 reads in T vs 946 reads in TO), OTU8 (3 reads in T vs 601 reads in TO), OTU24 (0 reads in T vs 155 reads in TO), OTU19 (1 read in T vs 166 reads in TO), OTU14 (3 reads in T vs 251 reads in TO), and OTU15 (3 reads in T vs 187 reads in TO) (Figure 4A). Taxonomically, these OTUs were assigned to the genera *Paenarthrobacter, Streptomyces, Microcoleus, Tychonema, Micromonospora*, and *Acidovorax*, respectively (Supplementary Table 2).

The most significantly depleted OTUs included OTU3 (11019 reads in T vs 302 reads in TO), OTU13 (2119 reads in T vs 302 reads in TO), OTU9 (866 reads in T vs 26 reads in TO), OTU5 (880 reads in T vs 3 reads in TO), and OTU7 (566 reads in T vs 6 reads in TO) (Figure 4B). Taxonomically, these OTUs were assigned to the genera *Flavobacterium, Pseudomonas, Massilia, Pedobacter*, and *Dyadobacter*, respectively (Supplementary Table 3).

## 4 Discussion

In the present study, tomato/potato-onion intercropping significantly increased alpha diversity indices (Shannon, Chao1, and ACE) of AMF spore-associated bacterial communities compared to monoculture, supporting our first research question. This diversity enhancement stems from the intricate resource dynamics and micro environmental regulation inherent in intercropping systems. Host plants allocate 4-20% of photosynthetic carbon to AMF as hexoses and fatty acids to support extraradical hyphal network expansion (Hodge, 1996; Rich et al., 2017; Zhang et al., 2022), while potato-onion root exudates—enriched in organic acids, phenolics, and carbohydrates—modify rhizosphere pH, nutrient bioavailability, and microbial niche partitioning (Linderman, 1988; Zhou et al., 2023). This dual input of plant-derived carbon and exudate-mediated microenvironmental tuning creates a more heterogeneous habitat, fostering higher bacterial diversity. Such a pattern aligns with broader ecological theory suggesting that increased plant diversity drives microbial diversity through resource partitioning (Van der Heijden et al., 2008), and is supported by empirical evidence that intercropping-induced root exudate complexity directly enhances rhizosphere microbial richness (Li et al., 2024). It is worth noting that our study was conducted under low-P conditions (which promote AMF colonization), so observed microbial shifts may also reflect P limitation effects.

Intercropping not only elevated diversity but also reshaped community composition, with *Actinobacteria, Cyanobacteria*, and functional genera (e.g., *Streptomyces, Rhodococcus, Paenarthrobacter*) significantly enriched. This taxonomic shift reflects niche specialization: AMF spores secrete chitin, lipids, and signaling molecules (Frey-Klett et al., 2007), creating a microhabitat that selects for bacteria with hydrolytic capabilities (e.g., chitinases, phosphatases) and symbiotic traits. Our detection of 12 dominant bacterial phyla (led by Proteobacteria, Bacteroidetes, Actinobacteriota, and Firmicutes) aligns with Zhang et al. (2022) characterization of core AMF-associated taxa, but extends this by identifying intercropping-specific enrichment of Actinobacteriota—a phylum critical for mediating AMF-plant mutualisms via secondary metabolite secretion and nutrient mobilization (Song et al., 2010; Wang et al., 2023). Specifically, *Streptomyces*—a dominant enriched genus—produces auxins and cellulases that may enhance AMF spore germination and hyphal growth (Jin et al., 2024; Sadeghi et al., 2012; Chater, 2016), while *Rhodococcus* and *Bacillus* likely promote AMF root colonization by modulating hyphal branching and symbiotic signaling (Battini et al., 2016; Lecomte et al., 2011), suggesting potential plant-AMF-bacteria synergies. This observation of intercropping-induced enrichment of functionally beneficial bacterial taxa supports our second research question. Additionally, since the F338/R806 primer pair may introduce amplification bias against specific phyla (e.g., Verrucomicrobia), multiple factors should be considered when analyzing low-abundance phyla.

These community shifts could have notable functional implications. Enriched taxa in intercropping systems are linked to key agroecosystem functions based on their well-documented traits in prior studies: *Paenarthrobacter* solubilizes inorganic phosphorus to improve plant uptake (Salimi et al., 2023); *Janthinobacterium* produces antifungal compounds that suppress soil-borne pathogens (Haack et al., 2016); and *Cyanobacteria* contribute to nitrogen fixation, enhancing soil fertility (Álvarez et al., 2023). This functional coordination suggests intercropping assembles a microbiome with enhanced nutrient cycling and plant-beneficial traits, rather than inducing random taxonomic shifts. Our identification of 137 upregulated OTUs (predominantly Proteobacteria/Actinobacteriota) and 19 depleted OTUs (Bacteroidetes/Proteobacteria) mirrors Gao D. et al. (2021) observation of intercropping-driven taxonomic specificity, reinforcing that plant diversity selects for functionally complementary microbial consortia. This aligns with the “functional redundancy” hypothesis, where higher diversity ensures stable ecosystem function through overlapping taxa roles (Allison et al., 2008). However, we note that functional redundancy was not directly measured in this study—our inference is based on taxonomic diversity patterns and prior literature, not functional assays.

Beyond productivity, these spore-associated bacteria may play underappreciated roles in stress resilience. AMF are known to mitigate heavy metal toxicity and drought stress via immobilization and osmotic regulation (Wipf et al., 2019), and their associated bacteria could hypothetically synergistically reinforce these stress-mitigating traits, drawing on prior findings regarding related bacterial taxa: *Streptomyces* species have been reported to degrade organic pollutants and produce siderophores to alleviate metal stress (Dimkpa et al., 2009), while *Allorhizobium* has been shown to modulate plant stress hormone signaling (e.g., abscisic acid, ABA) to improve drought tolerance. However, the mechanistic links between intercropping practices, spore-associated bacterial activity, and plant stress tolerance have not been examined in the present study and thus remain underexplored—particularly in intensive agricultural systems where soil degradation and abiotic stress are widespread (Chen, 2022), rendering this a priority for future investigation.

Despite these insights, limitations exist. Our study focuses on community structure rather than direct functional validation; future work should employ gnotobiotic systems to quantify how key taxa (e.g., *Streptomyces, Rhodococcus*) affect AMF spore viability, hyphal growth, and plant nutrient uptake. Additionally, greenhouse conditions may not fully replicate field-scale variability in soil type and climate, necessitating field trials to confirm the generalizability of our findings.

In conclusion, this study demonstrates that tomato/potato-onion intercropping reshapes AMF spore-associated bacterial communities toward higher diversity and functionally beneficial taxa. These findings advance our understanding of how cropping systems modulate microbial symbioses and provide a mechanistic foundation for harnessing AMF-bacterial interactions to improve sustainable agroecosystem management.

## 5 Conclusion

In summary, tomato/potato-onion intercropping significantly enhances the diversity of AMF spore-associated bacterial communities in the tomato rhizosphere, enriches beneficial taxa (e.g., *Actinobacteria*) and functional genera (e.g., *Streptomyces, Rhodococcus*), and modifies community composition relative to monoculture, providing foundational data on intercropping-driven microbial dynamics. Mechanistic links between these bacterial shifts, plant-microbe interactions, and key functions (e.g., nutrient cycling, stress tolerance) remain unresolved, requiring future functional validation of enriched taxa. Specifically, verifying their roles in AMF spore germination, nutrient acquisition, and stress mitigation could support translating microbial shifts into strategies for sustainable agriculture.

## Data Availability

The data presented in this study are publicly available. The data can be found here: https://www.ncbi.nlm.nih.gov/bioproject, accession PRJNA1291378.
