# Supplementary material for: Intercropping with potato-onion alters arbuscular mycorrhizal fungi spore-associated bacterial communities of tomato rhizosphere
Source: Front Microbiol. 2025 Nov 26;16:1686962. doi: 10.3389/fmicb.2025.1686962 (PMC12690609; doi:10.3389/fmicb.2025.1686962)
Supplement: Supplementary file 1 [file Table_1.docx]

**Table S1.** Relative abundances (%) of the top classified 50 bacterial genera detected for different treatments with tomato monoculture system (T) and tomato/potato-onion intercropping system (TO). *, **, and *** indicate significant difference at *p* < 0.05, *p* < 0.01, and *p* < 0.001 (Student’s t-test), respectively.

| genus | T | TO | P value |
| --- | --- | --- | --- |
| *Janthinobacterium* | 37.68±1.88 | 43.87±0.65 | 0.006** |
| *Pseudomonas* | 29.65±2.32 | 18.10±0.77 | 0.001*** |
| *Flavobacterium* | 25.22±2.74 | 0.89±0.03 | 0.000*** |
| *Rhodococcus* | 1.21±0.16 | 11.51±0.57 | 0.000*** |
| *Paenarthrobacter* | 0.02±0.00 | 2.57±0.12 | 0.000*** |
| *Pedobacter* | 2.12±0.37 | 0.27±0.04 | 0.001*** |
| *Massilia* | 1.99±0.05 | 0.21±0.03 | 0.000*** |
| *Streptomyces* | 0.01±0.01 | 1.62±0.12 | 0.000*** |
| *Dyadobacter* | 1.31±0.08 | 0.13±0.02 | 0.000*** |
| *Sphingomonas* | 0.03±0.01 | 1.16±0.25 | 0.000*** |
| *Microbacterium* | 0.03±0.01 | 0.86±0.08 | 0.000*** |
| *unclassified_Chloroplast* | 0.01±0.01 | 0.87±0.07 | 0.000*** |
| *Phormidium_IAM_M-71* | 0.02±0.01 | 0.84±0.14 | 0.000*** |
| *Tychonema_CCAP_1459-11B* | 0.06±0.02 | 0.78±0.27 | 0.01*** |
| *Bacillus* | 0.00±0.00 | 0.33±0.12 | 0.01*** |
| *Aeromicrobium* | 0.02±0.00 | 0.69±0.27 | 0.000*** |
| *Micromonospora* | 0.01±0.00 | 0.69±0.01 | 0.000*** |
| *Acidovorax* | 0.01±0.00 | 0.63±0.29 | 0.000*** |
| *Allorhizobium* | 0.04±0.01 | 0.50±0.32 | 0.000*** |
| *Microcoleus_PCC-7113* | 0.00±0.00 | 0.42±0.06 | 0.000*** |
| *Staphylococcus* | 0.00±0.00 | 0.09±0.01 | 0.000*** |
| *Nocardioides* | 0.01±0.00 | 0.34±0.07 | 0.000*** |
| *Marmoricola* | 0.01±0.00 | 0.35±0.14 | 0.000*** |
| *Haliangium* | 0.02±0.00 | 0.33±0.01 | 0.000*** |
| *metagenome* | 0.00±0.00 | 0.33±0.03 | 0.000*** |
| *Devosia* | 0.01±0.02 | 0.31±0.04 | 0.000*** |
| *Sphingobium* | 0.01±0.00 | 0.31±0.06 | 0.001*** |
| *unclassified_Ardenticatenaceae* | 0.01±0.01 | 0.28±0.03 | 0.000*** |
| *unclassified_Gemmatimonadaceae* | 0.01±0.00 | 0.24±0.03 | 0.000*** |
| *Archangium* | 0.01±0.00 | 0.23±0.01 | 0.000*** |
| *Galbitalea* | 0.03±0.04 | 0.18±0.03 | 0.007** |
| *Lysobacter* | 0.00±0.00 | 0.21±0.02 | 0.000*** |
| *Lechevalieria* | 0.00±0.00 | 0.20±0.05 | 0.003** |
| *unclassified_Microscillaceae* | 0.00±0.00 | 0.18±0.00 | 0.000*** |
| *Cystobacter* | 0.00±0.00 | 0.18±0.02 | 0.000*** |
| *unclassified_Polyangiaceae* | 0.00±0.00 | 0.18±0.01 | 0.000*** |
| *Ohtaekwangia* | 0.00±0.00 | 0.18±0.01 | 0.000*** |
| *unclassified_Acidobacteria_Subgroup_6* | 0.00±0.00 | 0.18±0.03 | 0.000*** |
| *Pseudolabrys* | 0.00±0.00 | 0.17±0.02 | 0.000*** |
| *Steroidobacter* | 0.00±0.00 | 0.17±0.03 | 0.000*** |
| *Altererythrobacter* | 0.00±0.00 | 0.16±0.06 | 0.009** |
| *Arthrobacter* | 0.16±0.05 | 0.00±0.00 | 0.000*** |
| *Synechococcus_IR11* | 0.00±0.00 | 0.15±0.02 | 0.000*** |
| *Variovorax* | 0.03±0.01 | 0.11±0.00 | 0.000*** |
| *Pseudoxanthomonas* | 0.01±0.00 | 0.14±0.03 | 0.003** |
| *Brevundimonas* | 0.00±0.00 | 0.14±0.47 | 0.007** |
| *Aliterella_CENA595* | 0.00±0.00 | 0.14±0.07 | 0.022* |
| *Comamonas* | 0.00±0.00 | 0.14±0.01 | 0.000*** |
| *Ensifer* | 0.00±0.00 | 0.13±0.01 | 0.000*** |
| *Phycicoccus* | 0.01±0.00 | 0.12±0.01 | 0.000*** |

**Table S2.** The OTUs enriched in the cropping system treatment. T:tomato monoculture system, TO:tomato/potato-onion intercropping system.

| phylum | genus | otu_id | T | TO |
| --- | --- | --- | --- | --- |
| Actinobacteria | *Paenarthrobacter* | OTU6 | 0.017 | 2.570 |
| Actinobacteria | *Streptomyces* | OTU8 | 0.006 | 1.613 |
| Cyanobacteria | *Microcoleus_PCC-7113* | OTU24 | 0.000 | 0.420 |
| Cyanobacteria | *Tychonema_CCAP_1459-11B* | OTU19 | 0.002 | 0.448 |
| Actinobacteria | *Micromonospora* | OTU14 | 0.007 | 0.688 |
| Cyanobacteria | *unclassified_Chloroplast* | OTU12 | 0.008 | 0.746 |
| Proteobacteria | *Acidovorax* | OTU15 | 0.007 | 0.512 |
| Proteobacteria | *Pseudomonas* | OTU20 | 0.001 | 0.272 |
| Cyanobacteria | *Phormidium_IAM_M-71* | OTU10 | 0.017 | 0.811 |
| Proteobacteria | *Comamonas* | OTU83 | 0.000 | 0.142 |
| Proteobacteria | *Ensifer* | OTU398 | 0.000 | 0.127 |
| Proteobacteria | *unclassified_Polyangiaceae* | OTU45 | 0.001 | 0.181 |
| Cyanobacteria | *unclassified_Chloroplast* | OTU34 | 0.000 | 0.127 |
| Actinobacteria | *Marmoricola* | OTU135 | 0.005 | 0.351 |
| Proteobacteria | *Massilia* | OTU247 | 0.000 | 0.127 |
| Proteobacteria | *Acidovorax* | OTU291 | 0.000 | 0.117 |
| Actinobacteria | *Lechevalieria* | OTU46 | 0.001 | 0.197 |
| Proteobacteria | *Sphingomonas* | OTU26 | 0.004 | 0.290 |
| Proteobacteria | *Sphingomonas* | OTU27 | 0.013 | 0.648 |
| Chloroflexi | *unclassified_Ardenticatenaceae* | OTU33 | 0.000 | 0.101 |
| Proteobacteria | *Devosia* | OTU68 | 0.000 | 0.105 |
| Cyanobacteria | *Synechococcus_IR11* | OTU41 | 0.001 | 0.148 |
| Proteobacteria | *Polyangium* | OTU50 | 0.000 | 0.095 |
| Actinobacteria | *Aeromicrobium* | OTU16 | 0.020 | 0.685 |
| Proteobacteria | *Steroidobacter* | OTU142 | 0.000 | 0.003 |
| Firmicutes | *Staphylococcus* | OTU42 | 0.000 | 0.084 |
| Proteobacteria | *Acidibacter* | OTU295 | 0.000 | 0.079 |
| Firmicutes | *Paenisporosarcina* | OTU61 | 0.000 | 0.077 |
| Actinobacteria | *Nocardioides* | OTU154 | 0.000 | 0.077 |
| Actinobacteria | *Crossiella* | OTU49 | 0.000 | 0.071 |
| Proteobacteria | *Pseudolabrys* | OTU102 | 0.000 | 0.075 |
| Bacteroidetes | *Ohtaekwangia* | OTU47 | 0.000 | 0.070 |
| Proteobacteria | *Bradyrhizobium* | OTU57 | 0.001 | 0.110 |
| Proteobacteria | *Altererythrobacter* | OTU70 | 0.000 | 0.061 |
| Chloroflexi | *unclassified_Ardenticatenaceae* | OTU190 | 0.000 | 0.059 |
| Proteobacteria | *Physcomitrella_patens* | OTU59 | 0.000 | 0.066 |
| Cyanobacteria | *Ancylothrix_8PC* | OTU63 | 0.000 | 0.057 |
| Proteobacteria | *unclassified_Methyloligellaceae* | OTU130 | 0.000 | 0.059 |
| Actinobacteria | *metagenome* | OTU66 | 0.000 | 0.061 |
| Proteobacteria | *Pedomicrobium* | OTU203 | 0.000 | 0.058 |
| Acidobacteria | *unclassified_Acidobacteria_Subgroup_6* | OTU235 | 0.001 | 0.088 |
| Firmicutes | *Bacillus* | OTU21 | 0.001 | 0.425 |
| Proteobacteria | *metagenome* | OTU123 | 0.000 | 0.057 |
| Chloroflexi | *metagenome* | OTU147 | 0.001 | 0.089 |
| Cyanobacteria | *Coleofasciculus_CCY0602* | OTU56 | 0.001 | 0.091 |
| Proteobacteria | *Sphingomonas* | OTU95 | 0.002 | 0.140 |
| Proteobacteria | *Paracoccus* | OTU136 | 0.000 | 0.052 |
| Actinobacteria | *Microbacterium* | OTU17 | 0.027 | 0.861 |
| Proteobacteria | *Haliangium* | OTU114 | 0.001 | 0.088 |
| Bacteroidetes | *Pedobacter* | OTU84 | 0.000 | 0.052 |
| Proteobacteria | *metagenome* | OTU103 | 0.000 | 0.050 |
| Proteobacteria | *Enterobacter* | OTU171 | 0.000 | 0.116 |
| Proteobacteria | *Aminobacter* | OTU132 | 0.002 | 0.117 |
| Proteobacteria | *Altererythrobacter* | OTU246 | 0.001 | 0.095 |
| Proteobacteria | *Sphingobium* | OTU38 | 0.003 | 0.147 |
| Gemmatimonadetes | *unclassified_Gemmatimonadaceae* | OTU196 | 0.000 | 0.045 |
| Bacteroidetes | *Flavobacterium* | OTU74 | 0.000 | 0.049 |
| Proteobacteria | *Acinetobacter* | OTU73 | 0.000 | 0.064 |
| Firmicutes | *Bacillus* | OTU51 | 0.000 | 0.102 |
| Proteobacteria | *Steroidobacter* | OTU191 | 0.001 | 0.073 |
| Actinobacteria | *Nocardioides* | OTU55 | 0.001 | 0.072 |
| Proteobacteria | *unclassified_Xanthobacteraceae* | OTU411 | 0.000 | 0.041 |
| Bacteroidetes | *unclassified_Amoebophilaceae* | OTU65 | 0.000 | 0.044 |
| Proteobacteria | *Hyphomicrobium* | OTU187 | 0.000 | 0.043 |
| Firmicutes | *Bacillus* | OTU37 | 0.001 | 0.197 |
| Actinobacteria | *unclassified_Gaiellales* | OTU244 | 0.000 | 0.044 |
| Proteobacteria | *Sphingobium* | OTU64 | 0.000 | 0.045 |
| Proteobacteria | *Cystobacter* | OTU23 | 0.007 | 0.231 |
| Proteobacteria | *Archangium* | OTU32 | 0.007 | 0.231 |
| Proteobacteria | *Sphingobium* | OTU28 | 0.002 | 0.114 |
| Proteobacteria | *Dongia* | OTU274 | 0.000 | 0.039 |
| Cyanobacteria | *Nostoc_PCC-7107* | OTU75 | 0.000 | 0.047 |
| Actinobacteria | *Gaiella* | OTU234 | 0.000 | 0.038 |
| Proteobacteria | *Novosphingobium* | OTU81 | 0.002 | 0.105 |
| Proteobacteria | *Devosia* | OTU40 | 0.006 | 0.204 |
| Chloroflexi | *unclassified_AKIW781* | OTU255 | 0.000 | 0.035 |
| Proteobacteria | *Luteimonas* | OTU62 | 0.000 | 0.034 |
| Firmicutes | *Clostridium_sensu_stricto_12* | OTU72 | 0.000 | 0.044 |
| Proteobacteria | *Cellvibrio* | OTU89 | 0.002 | 0.099 |
| Proteobacteria | *Stenotrophomonas* | OTU186 | 0.000 | 0.038 |
| Actinobacteria | *Nocardioides* | OTU259 | 0.000 | 0.035 |
| Proteobacteria | *Lysobacter* | OTU188 | 0.000 | 0.031 |
| Cyanobacteria | *Leptolyngbya_ANT.L52.2* | OTU82 | 0.000 | 0.032 |
| Cyanobacteria | *Oscillatoria_PCC-6304* | OTU106 | 0.000 | 0.033 |
| Actinobacteria | *Ilumatobacter* | OTU211 | 0.000 | 0.033 |
| Gemmatimonadetes | *unclassified_Gemmatimonadaceae* | OTU231 | 0.000 | 0.031 |
| Actinobacteria | *Cutibacterium* | OTU98 | 0.000 | 0.030 |
| Cyanobacteria | *Aerosakkonema_Lao26* | OTU133 | 0.001 | 0.081 |
| Proteobacteria | *unclassified_Rhodanobacteraceae* | OTU266 | 0.000 | 0.036 |
| Cyanobacteria | *Aliterella_CENA595* | OTU35 | 0.003 | 0.140 |
| Proteobacteria | *Haliangium* | OTU206 | 0.000 | 0.030 |
| Acidobacteria | *unclassified_Acidobacteria_Subgroup_6* | OTU387 | 0.000 | 0.030 |
| Bacteroidetes | *Ohtaekwangia* | OTU69 | 0.000 | 0.031 |
| Proteobacteria | *unclassified_Xanthobacteraceae* | OTU129 | 0.000 | 0.027 |
| Proteobacteria | *Bosea* | OTU293 | 0.000 | 0.029 |
| Bacteroidetes | *unclassified_Microscillaceae* | OTU113 | 0.001 | 0.056 |
| Proteobacteria | *Rubrivivax* | OTU200 | 0.000 | 0.030 |
| Proteobacteria | *Ellin6067* | OTU120 | 0.000 | 0.025 |
| Proteobacteria | *Skermanella* | OTU222 | 0.000 | 0.026 |
| Gemmatimonadetes | *unclassified_Gemmatimonadaceae* | OTU58 | 0.001 | 0.051 |
| Bacteroidetes | *Lacibacter* | OTU78 | 0.000 | 0.028 |
| Proteobacteria | *Noviherbaspirillum* | OTU160 | 0.000 | 0.026 |
| Gemmatimonadetes | *unclassified_AKAU4049* | OTU329 | 0.000 | 0.024 |
| Proteobacteria | *unclassified_Moraxellaceae* | OTU437 | 0.000 | 0.026 |
| Actinobacteria | *Rhodococcus* | OTU210 | 0.000 | 0.024 |
| Actinobacteria | *Blastococcus* | OTU362 | 0.000 | 0.027 |
| Proteobacteria | *unclassified_Moraxellaceae* | OTU104 | 0.000 | 0.028 |
| Firmicutes | *Lactobacillus* | OTU143 | 0.000 | 0.032 |
| Cyanobacteria | *Tychonema_CCAP_1459-11B* | OTU18 | 0.005 | 0.273 |
| Bacteroidetes | *Ohtaekwangia* | OTU151 | 0.000 | 0.024 |
| Bacteroidetes | *Niastella* | OTU313 | 0.000 | 0.024 |
| Proteobacteria | *Rheinheimera* | OTU205 | 0.000 | 0.025 |
| Bacteroidetes | *unclassified_Microscillaceae* | OTU52 | 0.003 | 0.097 |
| Firmicutes | *Caldicoprobacter* | OTU290 | 0.000 | 0.047 |
| Proteobacteria | *IS-44* | OTU265 | 0.000 | 0.022 |
| Proteobacteria | *Defluviicoccus* | OTU139 | 0.000 | 0.022 |
| Firmicutes | *Bacillus* | OTU429 | 0.000 | 0.022 |
| Proteobacteria | *Lysobacter* | OTU54 | 0.001 | 0.068 |
| Proteobacteria | *Dyella* | OTU175 | 0.000 | 0.021 |
| Actinobacteria | *CL500-29_marine_group* | OTU396 | 0.000 | 0.023 |
| Proteobacteria | *Thermomonas* | OTU71 | 0.002 | 0.062 |
| Patescibacteria | *unclassified_Saccharimonadales* | OTU111 | 0.000 | 0.022 |
| Proteobacteria | *Pseudorhodoplanes* | OTU338 | 0.000 | 0.022 |
| Proteobacteria | *Hirschia* | OTU170 | 0.000 | 0.023 |
| Proteobacteria | *Legionella* | OTU299 | 0.000 | 0.023 |
| Actinobacteria | *Agromyces* | OTU195 | 0.001 | 0.044 |
| Bacteroidetes | *Niastella* | OTU112 | 0.000 | 0.020 |
| Bacteroidetes | *unclassified_Microscillaceae* | OTU149 | 0.000 | 0.020 |
| Patescibacteria | *Candidatus* | OTU199 | 0.000 | 0.022 |
| Proteobacteria | *unclassified_Alphaproteobacteria* | OTU125 | 0.000 | 0.020 |
| Proteobacteria | *Haliangium* | OTU319 | 0.000 | 0.021 |
| Actinobacteria | *Solirubrobacter* | OTU379 | 0.000 | 0.019 |
| Proteobacteria | *Pseudolabrys* | OTU122 | 0.003 | 0.096 |
| Proteobacteria | *Methyloceanibacter* | OTU134 | 0.001 | 0.045 |
| Proteobacteria | *Sandaracinus* | OTU131 | 0.000 | 0.019 |
| Proteobacteria | *Cupriavidus* | OTU183 | 0.000 | 0.020 |
| Proteobacteria | *unclassified_Clade_III* | OTU107 | 0.000 | 0.028 |

**Table S3.** The OTUs depleted in the cropping system treatment. T:tomato monoculture system, TO:tomato/potato-onion intercropping system.

| phylum | genus | otu_id | T | TO |
| --- | --- | --- | --- | --- |
| Proteobacteria | *Janthinobacterium* | OTU1 | 37.677 | 43.871 |
| Proteobacteria | *Pseudomonas* | OTU2 | 24.731 | 17.540 |
| Bacteroidetes | *Flavobacterium* | OTU3 | 25.218 | 0.834 |
| Actinobacteria | *Rhodococcus* | OTU11 | 0.244 | 0.508 |
| Proteobacteria | *Pseudomonas* | OTU13 | 4.914 | 0.281 |
| Bacteroidetes | *Pedobacter* | OTU29 | 0.119 | 0.203 |
| Actinobacteria | *Galbitalea* | OTU67 | 0.090 | 0.121 |
| Proteobacteria | *Variovorax* | OTU48 | 0.035 | 0.113 |
| Proteobacteria | *Massilia* | OTU9 | 1.987 | 0.074 |
| Proteobacteria | *zobium* | OTU101 | 0.021 | 0.071 |
| Proteobacteria | *Noviherbaspirillum* | OTU85 | 0.027 | 0.065 |
| Cyanobacteria | *Tychonema_CCAP_1459-11B* | OTU31 | 0.052 | 0.059 |
| Proteobacteria | *Kaistia* | OTU93 | 0.017 | 0.039 |
| Bacteroidetes | *Dyadobacter* | OTU7 | 1.293 | 0.013 |
| Bacteroidetes | *Chryseobacterium* | OTU140 | 0.007 | 0.010 |
| Bacteroidetes | *Pedobacter* | OTU5 | 1.992 | 0.008 |
| Bacteroidetes | *Chryseobacterium* | OTU215 | 0.003 | 0.002 |
| Actinobacteria | *Arthrobacter* | OTU22 | 0.159 | 0.001 |
| Bacteroidetes | *Pedobacter* | OTU216 | 0.005 | 0.000 |
